# A Modified R-Type Bacteriocin Specifically Targeting Clostridium difficile Prevents Colonization of Mice without Affecting Gut Microbiota Diversity

**DOI:** 10.1128/mBio.02368-14

**Published:** 2015-03-24

**Authors:** Dana Gebhart, Stephen Lok, Simon Clare, Myreen Tomas, Mark Stares, Dean Scholl, Curtis J. Donskey, Trevor D. Lawley, Gregory R. Govoni

**Affiliations:** ^a^ AvidBiotics Corp., South San Francisco, California, USA; ^b^ Microbial Pathogenesis Laboratory, Wellcome Trust Sanger Institute, Hinxton, United Kingdom; ^c^ Geriatric Research Education and Clinical Center, Louis Stokes Cleveland Department of Veterans Affairs Medical Center, Cleveland, Ohio, USA; ^d^ Division of Infectious Diseases & HIV Medicine, Department of Medicine, Case Western Reserve University School of Medicine, Cleveland, Ohio, USA; ^e^ Host Microbiota Interactions Laboratory, Wellcome Trust Sanger Institute, Hinxton, United Kingdome; University of Nebraska

## Abstract

Clostridium difficile is a leading cause of nosocomial infections worldwide and has become an urgent public health threat requiring immediate attention. Epidemic lineages of the BI/NAP1/027 strain type have emerged and spread through health care systems across the globe over the past decade. Limiting person-to-person transmission and eradicating C. difficile, especially the BI/NAP1/027 strain type, from health care facilities are difficult due to the abundant shedding of spores that are impervious to most interventions. Effective prophylaxis for C. difficile infection (CDI) is lacking. We have genetically modified a contractile R-type bacteriocin (“diffocin”) from C. difficile strain CD4 to kill BI/NAP1/027-type strains for this purpose. The natural receptor binding protein (RBP) responsible for diffocin targeting was replaced with a newly discovered RBP identified within a prophage of a BI/NAP1/027-type target strain by genome mining. The resulting modified diffocins (a.k.a. Avidocin-CDs), Av-CD291.1 and Av-CD291.2, were stable and killed all 16 tested BI/NAP1/027-type strains. Av-CD291.2 administered in drinking water survived passage through the mouse gastrointestinal (GI) tract, did not detectably alter the mouse gut microbiota or disrupt natural colonization resistance to C. difficile or the vancomycin-resistant Enterococcus faecium (VREF), and prevented antibiotic-induced colonization of mice inoculated with BI/NAP1/027-type spores. Given the high incidence and virulence of the pathogen, preventing colonization by BI/NAP1/027-type strains and limiting their transmission could significantly reduce the occurrence of the most severe CDIs. This modified diffocin represents a prototype of an Avidocin-CD platform capable of producing targetable, precision anti-C. difficile agents that can prevent and potentially treat CDIs without disrupting protective indigenous microbiota.

## IMPORTANCE

Treatment and prevention strategies for bacterial diseases rely heavily on traditional antibiotics, which impose strong selection for resistance and disrupt protective microbiota. One consequence has been an upsurge of opportunistic pathogens, such as Clostridium difficile, that exploit antibiotic-induced disruptions in gut microbiota to proliferate and cause life-threatening diseases. We have developed alternative agents that utilize contractile bactericidal protein complexes (R-type bacteriocins) to kill specific C. difficile pathogens. Efficacy in a preclinical animal study indicates these molecules warrant further development as potential prophylactic agents to prevent C. difficile infections in humans. Since these agents do not detectably alter the indigenous gut microbiota or colonization resistance in mice, we believe they will be safe to administer as a prophylactic to block transmission in high-risk environments without rendering patients susceptible to enteric infection after cessation of treatment.

## INTRODUCTION

Clostridium difficile infection (CDI) represents a significant health risk, particularly to an aging population. The U.S. Centers for Disease Control and Prevention considers this pathogen to be a major public health threat requiring urgent attention (1). An estimated 250,000 hospitalizations and 14,000 deaths per year are caused by CDIs in the United States alone (1). Recent studies indicate 30 to 35% of North American CDIs were due to BI/NAP1/027-type (ribotype 027 [RT027]) strains (2, 3), most of which are clonal and derived from 1 of 2 epidemic lineages (FQR1 and FQR2) that independently acquired fluoroquinolone resistance (4). Recent studies have demonstrated a clear association between colonization with RT027 strains and a more severe infection outcome (2). Antibiotic treatment options for CDI, particularly those caused by RT027 strains, are plagued by high rates of relapse or recurrence after successful initial treatment (5, 6). Because today’s preventative options are limited to antibiotic stewardship and good hygienic practices (7, 8), new preventative approaches are urgently needed. Current approaches to combat infectious diseases do not often consider the off-target effects antibiotics have on healthy microbiota. The unintended loss of a diverse and stable gut microbiota has a dramatic detrimental impact on colonization resistance to opportunistic pathogens, such as C. difficile, and other gut metabolism-related issues (9–13). Effective and long-lasting protection from these opportunistic pathogens requires maintaining or restoring the healthy diversity of the gut microbiota.

A series of high-molecular-weight R-type bacteriocins termed “diffocins,” which originate from and specifically kill C. difficile, were recently isolated (14). Diffocins are analogous to R-type “pyocins” from Pseudomonas aeruginosa and contain contractile myophage-like sheath structures coupled to receptor binding proteins (RBPs) via tail fibers and a baseplate (see Fig. S1 in the supplemental material) (14–16). The RBPs serve as targeting proteins and determine a bacteriocin’s killing specificity by binding unique cell-surface receptors on a target bacterium. High sequence variability between RBP genes among strains of the same species accounts for killing spectrum differences within each series of naturally produced R-type bacteriocins. Initiation of the bacteriocin killing process occurs upon binding of the RBP to its cognate receptor on the target bacterium. Surface binding triggers the sheath to contract and drive a needle-like core through the target cell’s envelope to create a small pore that dissipates membrane potential and promptly kills the bacterium without releasing cytoplasmic toxin (17). Although the binding is highly specific, the killing mechanism is generic and extremely potent; a single R-type bacteriocin is sufficient to kill a target bacterium (18). The quick, highly specific killing mechanism of diffocins makes them potential prophylactic agents for preventing CDI.

For production purposes, diffocin gene clusters from several C. difficile strains were cloned and expressed in Bacillus subtilis strain 168 (14). As in C. difficile, diffocin expression was inducible via initiation of an SOS response (14). While being prepared for preclinical studies, several of the natural diffocins were found to lack desirable properties. Diffocin-43593, which kills RT027 strains, and diffocin-16 were unstable when stored at 4°C; diffocin-4 when administered orally was unable to survive transit through the mouse gastrointestinal (GI) tract. Exchanging RBPs between unstable and stable diffocins failed to produce a more stable diffocin with the desired specificity. An alternative source of RBPs was sought. Based on our experience modifying R-type pyocins (17, 19), we mined the genome of an RT027 strain and identified a novel C. difficile prophage RBP gene (*ptsM*) that when used to construct a modified diffocin could direct the scaffold of diffocin-4 to kill RT027 isolates regardless of phylogeny. Modified R-type bacteriocins, or Avidocin-CDs, constructed with PtsM and administered to mice in drinking water, were able to survive GI tract transit, prevent colonization of mice exposed to RT027 spores, and not detectably alter gut microbiota or colonization resistance. The data suggest that modified diffocins may serve as precision antibacterial proteins to prevent or treat CDIs in humans while preserving the important healthy diversity of their gut microbiota.

## RESULTS

### Retargeting diffocins using an RBP from a C. difficile prophage.

Given the predominance of RT027 strains in many locations and their association with a more severe disease phenotype (2), we focused on generating a stable diffocin to target these strains. Naturally occurring diffocin-43593 kills RT027 strains (14) but lacks stable activity when stored at 4°C (see Fig. S2 in the supplemental material). Analyses of the predicted diffocin structures determined that diffocin-43593 and diffocin-4, which is more stable but does not target RT027 strains, are practically identical except for their highly variable RBPs. Replacement of the RBP on diffocin-4 with the diffocin-43593 RBP generated a diffocin-4-based bacteriocin targeting RT027 strains, but it was not stable (data not shown). These physical property results (pH and temperature sensitivity profiles and survival in the GI tract) indicated that the natural ~200-kDa multidomain (~1,700 residues), flower-like RBP structures were inherently unstable. For example, exchanging the RBP on diffocin-4, which is stable, with the RBP from diffocin-43593 made the resulting bacteriocin fusion thermally unstable and more acid labile (data not shown). Accordingly, we sought another source of RBP genes. Reasoning that recently acquired prophages are likely to encode RBPs that bind extant surface receptors on their host cell, we pursued a genome mining approach, first described by Scholl et al. (19), in which the genomes of the intended target bacterial strains are screened for RBP genes within prophage insertions. This task was complicated by the fact that there was no identified C. difficile phage RBP, and published genomes for C. difficile phage do not contain homologues of natural diffocin RBPs.

**FIG 1:**
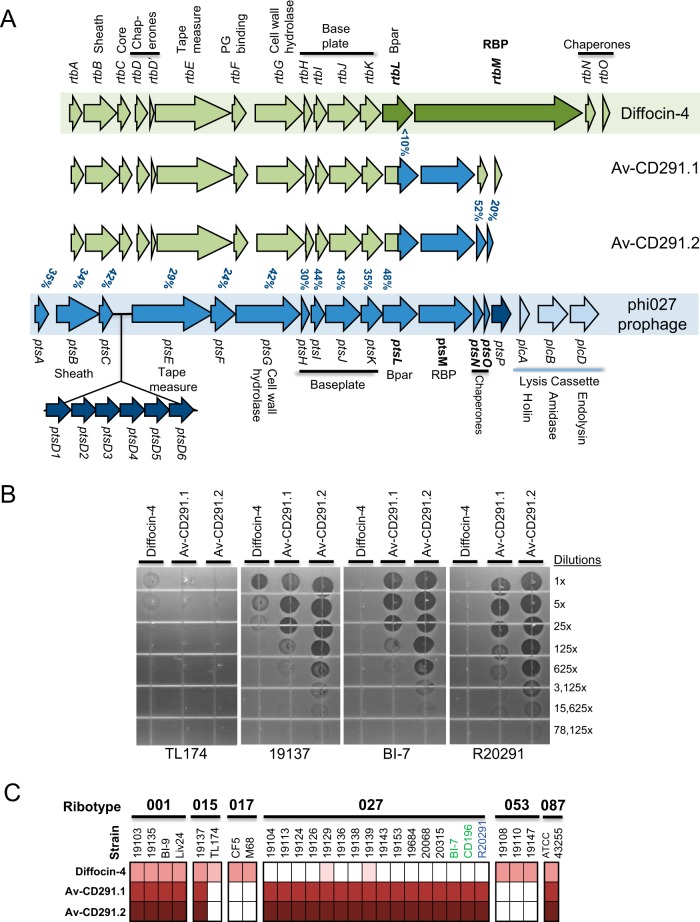
Retargeting diffocins with a prophage RBP from C. difficile strain R20291. (A) Schematic representation of gene clusters coding for diffocin-4 (green) and modified diffocins Av-CD291.1 and Av-CD291.2 and including the tail structure genes of the phi027 prophage (blue). Genes are color coded according to source. For the phi027 prophage, the lysis cassette present only in the phi027 prophage is depicted in light blue and structural genes with no homology in the diffocin gene cluster are depicted in dark blue. The percentages of similarity between the diffocin-4 and phi027 genes are given (blue). (B) *In vitro* spot bioassays for bactericidal activity are shown for several strains. Preparations of diffocin-4, Av-CD291.1, and Av-CD291.2 were serially diluted and spotted on a soft agar lawn containing the indicated target strain. Dark zones of clearance indicate killing. Overlapping but distinct killing specificities for each diffocin preparation, which were all produced from a genetically identical B. subtilis host cell and by the same method, indicate killing is specific to the diffocin and not due to any nonspecific, contaminating B. subtilis protein. (C) The strain coverage for diffocin-4, Av-CD291.1, and Av-CD291.2 for ribotypes 001, 015, 017, 027, 053, and 087 is shown. White indicates no killing, and maroon indicates killing—with intensity of maroon reflecting robustness of killing. Strain designations in green and blue indicate known FQR1 and FQR2 phylogenies, respectively. An additional 20 strains representing 13 ribotypes were also tested and found not to be sensitive to Av-CD291.2 (data not shown).

The genome sequence for strain R20291 (RT027) contains the phi027 prophage insertion (20). Sequence analyses determined that many structural genes in the prophage share nucleotide homology (24 to 52% similarity) with diffocin structural genes. This sequence similarity begins in the R-type bacteriocin A (*rtbA*) gene and extends into *rtbL*, the gene immediately upstream of the diffocin RBP (Fig. 1A). No homologue for the natural diffocin RBP (*rtbM*) in phi027 was found. To determine whether the prophage phage tail structure M gene (*ptsM*, open reading frame CD20291_1457) located in the same relative position as *rtbM* could serve the same receptor-binding function, we constructed a modified diffocin containing the prophage PtsM protein. Because diffocin baseplate attachment proteins, which are annotated as putative tail fibers, attach to RBPs and couple them to the baseplate, we surmised that modified diffocins with new RBPs would need to include the cognate baseplate attachment protein to function properly (14, 21). The phi027 and diffocin baseplate genes share only 30 to 44% homology (Fig. 1A), and thus, to make a functional agent the proximal portion of the diffocin-4 *rtbL* gene was fused to the distal portion of the phi027 prophage *ptsL* gene (Fig. 1A). The resulting modified diffocin, Avidocin-CD291.1 (Av-CD291.1), displayed bactericidal activity on vegetative forms of RT027 strains belonging to both the FQR1 and FQR2 lineages (4) (Fig. 1B and C). Because short genes immediately downstream of RBPs often encode chaperone proteins that assist in tail assembly (15, 21), the two short genes *ptsN* and *ptsO* downstream of *ptsM* were included in a second new construct (Fig. 1A). Preparations of the resulting diffocin, Av-CD291.2, had increased bactericidal activity relative to Av-CD291.1 (Fig. 1B). Importantly, both Av-CD291.1 and Av-CD291.2 killed all tested RT027 strains (*n* = 16) regardless of phylogeny (Fig. 1C) and remained robust after storage at 4°C (see Fig. S2 in the supplemental material). In addition to the RT027 strains tested, 40 isolates from 23 different ribotypes were screened for sensitivity. Other clinically significant ribotypes sensitive to Av-CD291.1 and Av-CD291.2 include ribotype 001 (4 of 4), ribotype 015 (1 of 2), ribotype 046 (*n* = 1), and the highly toxigenic ribotype 087 strain ATCC 43255 (also known as VPI 10463) (*n* = 1). Sequence homology searches (data not shown) identified *ptsM* homologues in the genomes of many other C. difficile isolates and bacteriophages, indicating that modified diffocins (Avidocin-CDs) constructed with other phage RBP variants may target other strain-types.

### Av-CD291.2 remains active during transit through the mouse GI tract.

**FIG 2:**
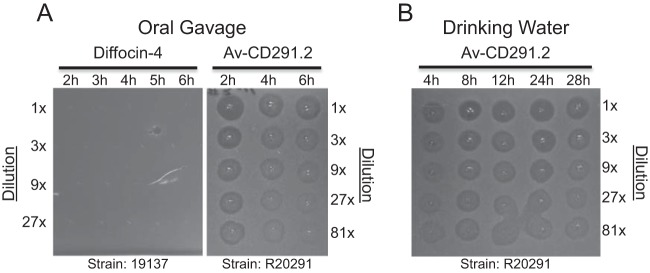
Enteric pharmacokinetics of diffocin-4 and Av-CD291.2 orally administered to mice. R-type bacteriocins that survive transit through the GI tract intact are detectable in feces by *in vitro* spot bioassays for bactericidal activity. Briefly, groups of 3 mice were administered diffocin-4 or Av-CD291.2 in 1% NaHCO_3_ (100 µg/dose [5 × 10^11^ KU]) via oral gavage (A) or in drinking water (60 µg/ml [3 × 10^11^ KU/ml]) containing 4% sucrose and 1% NaHCO_3_ for 28 h (B). Drinking water consumption averaged ~0.5 ml/h. Fecal pellets were collected and homogenized at the times indicated after oral gavage (A) or after introduction of Av-CD291.2 in drinking water (B). Fecal pellets collected at the indicated times were then filtered to remove microbial contaminants. The filtered homogenates were serially diluted and spotted on a soft agar lawn of the appropriately sensitive C. difficile strain R20291 (RT027). Dark zones of clearance indicate killing. Results from a representative mouse are shown for each condition.

Enteric pharmacokinetic studies were performed in mice orally administered natural diffocin and Avidocin-CD. Recovery of killing activity from fecal pellets was used as an indicator that active diffocin was present in the colon and had survived the full GI transit. In the initial experiments, single doses of either diffocin-4 or Av-CD291.2 (5 × 10^11^ killing units [KU], equivalent to ~100 µg) were administered to individual mice by oral gavage (Fig. 2A). A KU is the amount of agent needed to kill a single vegetative C. difficile cell; Avidocin-CDs do not affect the viability of C. difficile spores (see Fig. S3 in the supplemental material). Since both are acid labile below pH 3.5 to 4, each bacteriocin was formulated in a 1% sodium bicarbonate solution to buffer against stomach acidity, and an injection of ranitidine, a histamine H2-receptor antagonist that inhibits stomach acid production, was given prior to diffocin administration. No killing activity was observed in feces obtained from mice administered diffocin-4 (Fig. 2A). Additional experiments failed to detect diffocin-4 activity in cecal contents taken 2 h after diffocin administration (data not shown). In contrast, killing activity from Av-CD291.2 was detected in the feces at all time points taken for all mice (Fig. 2A). Recovery peaked 2 h after administration and tapered off over the next 4 h. In an effort to obtain more consistent dosing over a 24-h period, Av-CD291.2 was administered in the drinking water using a using a calibrated fluid administration device described by Bachmanov et al. (22). Sucrose (4% [wt/vol] final concentration) was added to the 1% bicarbonate formulation in order to increase water consumption and reduce diurnal variation in consumption rates (23). Killing activity from Av-CD291.2 was detected in the feces at all time points for all mice (Fig. 2B). Although a modest increase in the recovery of Av-CD291.2 killing activity was observed when mice were given ranitidine in the drinking water (data not shown), the increase was deemed insufficient to warrant its inclusion in subsequent studies.

### Prophylactic efficacy of Av-CD291.2 in a mouse model of C. difficile colonization.

We examined whether the modified diffocin could prevent colonization in an established mouse model of C. difficile spore transmission (24)*.* This model was designed and established to mimic the colonization of persons exposed to C. difficile spores in a typical contaminated environment, such as a health care facility, who have exposure levels well below a 100% infectious dose (ID_100_). In this study, healthy mice were exposed to contaminated cages containing an ID_90_ surface density of C. difficile spores (13 CFU/cm^2^) for 1.5 h before being individually housed in isolator cages and monitored for fecal shedding of C. difficile. By using spores from the clindamycin-resistant BI-7 strain (RT027), we were able to administer clindamycin after spore exposure to disrupt the healthy gut microbiota and its colonization resistance, yet not hinder BI-7 growth and super shedding (25). The Av-CD291.2 drinking water formulation used in Fig. 2B was administered to the treated cohorts (total *n* = 20) for 4 days, starting 4 h prior to spore exposure. Based on individual water consumption rates, the average daily Av-CD291.2 dose per mouse was 125 µg, or 6 mg/kg body weight. Placebo cohorts (total *n* = 20) received only excipient solution for the same duration. Each Av-CD291.2 and placebo cohort consisted of 10 male and 10 female mice.

The fecal shedding data indicate that Av-CD291.2 prevented BI-7 colonization completely. Fecal shedding of BI-7 was detected in all 10 male and 8 female mice in the placebo cohort (average of 7.5 × 10^7^ CFU/g of feces), for a colonization rate of 90% (Table 1; see Dataset S1 in the supplemental material). No fecal shedding of BI-7 was detected from any of the 20 Av-CD291.2-treated mice at the end of the 4-day treatment (limit of detection [LOD], 500 CFU/g feces) (Table 1; see Dataset S1). The BI-7 colonization rate rose to 95% on the third day after discontinuing the Av-CD291.2 treatment (Day 7; Table 1). This result was not unexpected since the severe microbiota disruption caused by clindamycin, and thereby loss of colonization resistance to C. difficile, can take 10 to 28 days to recover in a mouse (26). The Av-CD291.2-treated cohort received clindamycin for 2 days during the Av-CD291.2 treatment, and thus, these mice were still susceptible to C. difficile colonization after cessation of Av-CD291.2 treatment. Widespread, unintentional BI-7 contamination was also considered and deemed highly unlikely since none of the 4 sentinel mice serving as contamination monitors (and, therefore, not intentionally exposed to BI-7) became colonized with BI-7 during the course of the study. During the study, it was also noted that an indigenous, clindamycin-sensitive strain of C. difficile occasionally found in the resident mouse colony at the facility and unrelated to BI-7 was found in the feces of 45% of Av-CD291.2-treated mice and 50% of the sentinel mice at low levels (<2 × 10^4^ CFU/g feces; see Dataset S1). This strain has never been isolated from a mouse with acute disease, and there is no indication it had an effect on the clinical outcome of the present study. Next-generation DNA sequencing confirmed that it was a non-toxigenic, non-RT027 strain that does not encode the receptor for Av-CD291.2 binding (unpublished data). *In vitro* killing spot bioassays confirmed that this strain was insensitive to Av-CD291.2 and would not have been affected by Av-CD291.2 treatment. Previous studies have reported that non-toxigenic strains of C. difficile can block colonization of hamsters with RT027 strains (27). This phenomenon was not observed in this study since all 9 of the Av-CD291.2 treated mice shedding low levels of the nontoxigenic, indigenous clindamycin-sensitive strain became BI-7 super shedders after cessation of Av-CD291.2 treatment. The percentage of mice shedding the indigenous, clindamycin-sensitive strain in the placebo-treated groups was masked by the high level of BI-7 shedding (average of 7.5 × 10^7^ CFU/g of feces), which was more than 1,000 times higher than the levels observed for the clindamycin-sensitive strain in the Av-CD291.2-treated groups (<2 × 10^4^ CFU/g feces).

**TABLE 1:** Modified diffocin Av-CD291.2 prevents colonization in mice exposed to spores from C. difficile isolate BI/NAP1/027

Cohort	Results for^a^:					
	Day 4			Day 7^b^		
	Infected (*n*)	Total (*n*)	%	Infected (*n*)	Total (*n*)	%
Placebo						
Female	8	10	80	10	10	100
Male	10	10	100	10	10	100
**Total**	**18**	**20**	**90**	**20**	**20**	**100**
Av-CD291.2						
Female	0*	10	0*	9	10	90
Male	0**	10	0**	10	10	100
**Total**	0**	**20**	0**	**19**	**20**	**95**

^a^ Asterisks indicate statistically significant difference compared to the placebo control cohort by one-sided Fisher’s exact test: *, *P* < 0.001; **, *P* < 0.0001.

^b^ Three days after termination of treatment.

### Av-CD291.2 does not detectably alter the microbiome of the mouse gut.

The effective Av-CD291.2 dosing regimen from the prevention study was applied to normal healthy mice (*n* = 10) to determine what impact Av-CD291.2 had on the unperturbed mouse gut microbiome. The excipient solution (4% sucrose, 1% NaHCO_3_) was administered to mice as a placebo, negative control (*n* = 10). In order to confirm adequate sensitivity of the microbiota analyses, a cohort of mice (*n* = 10) was administered a low, sub-therapeutic dose of fidaxomicin (LD-fidaxomicin) in the same excipient solution. The average daily oral dose of fidaxomicin (0.8 mg/kg) was below the recommended 5-mg/kg human daily dose (28) and on the low end of the effective dose range observed in hamsters (29).

Next-generation sequencing of the V4 region of 16S rRNA genes was performed on DNA extracted from fecal samples collected pretreatment (day −1) and posttreatment (day +4) to monitor for changes in the gut microbial composition. A total of 2,996 operational taxonomic units (OTU) were detected in the study, with an average of 773 OTU per sample (see Dataset S2 in the supplemental material).

**FIG 3:**
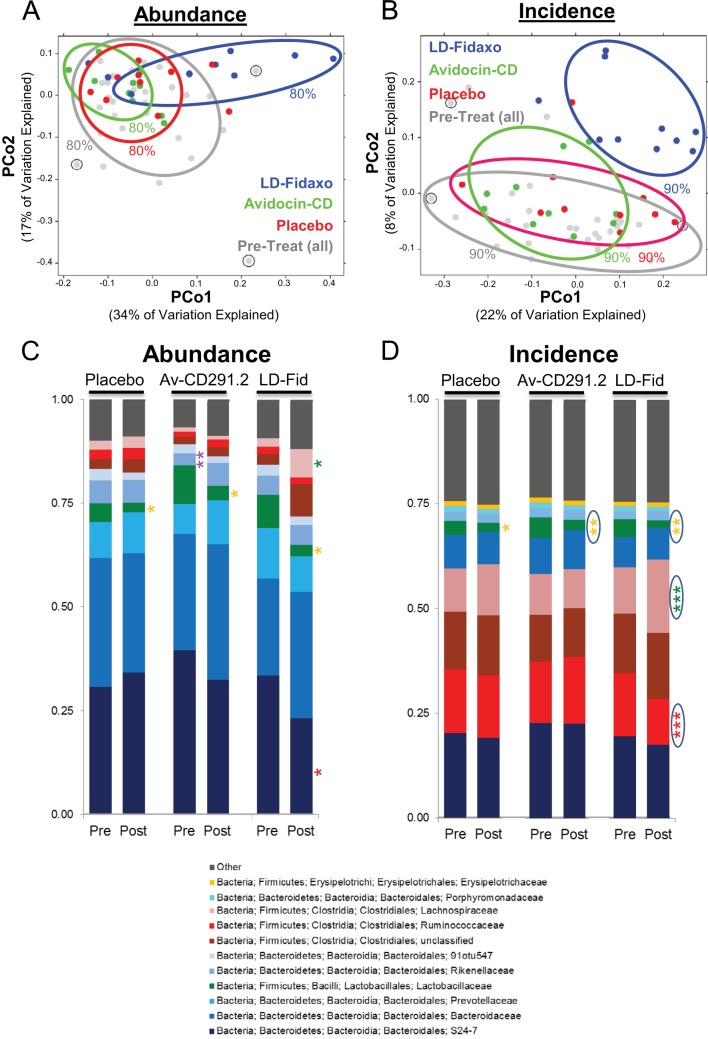
Oral administration of Av-CD291.2 in drinking water does not disturb the indigenous gut microbiota of healthy mice. (A) Analysis of the variance between microbial communities from the feces of healthy mice pretreatment (all) and posttreatment for each of the groups. The variance was assessed by the average relative abundance (weighted UniFrac distances) of OTU using principal component analysis. Samples are color coded according to treatment. Circled pretreatment samples indicate pretreatment outliers (one from each pretreatment cohort) that were removed from the microbiota analyses. Zone of inclusion are given with percentages. PCo1, principal component 1; PCo2, principal component 2. (B) Same as in panel A, except the average relative incidence (unweighted UniFrac distances) for OTU was assessed. (C) Bar charts depict the average family level composition (top 9) of OTU detected for each treatment group pre- and posttreatment by relative abundance. Significant differences between pre- and posttreatment are indicated by asterisks (*, 0.01 < *P* < 0.05; **, 0.001 < *P* < 0.01). Green asterisks indicate families elevated posttreatment, red asterisks indicate families reduced posttreatment, orange asterisks indicate families reduced posttreatment relative to all pretreatments, and purple indicates a family lower in one pretreatment cohort relative to most or all other pre- and posttreatment cohorts. (D) Same as in panel C, except the average family-level compositions (top 9) pre- and posttreatment were detected by relative incidence. Significant differences between pre- and posttreatment are indicated by asterisks as in panel C (***, *P* < 0.001). Circled asterisks indicate significant differences that remained after a false discovery rate correction was applied.

Multiple analyses failed to detect significant fecal microbiota changes in the animals exposed to Av-CD291.2. Initial alpha diversity analysis (number of OTU present) found no major disruptions posttreatment compared to pretreatment; a similar result was also observed for the LD-fidaxomicin cohort (see Fig. S4A in the supplemental material). For a more in-depth look, multiple beta diversity analyses (distribution of OTU) were also undertaken. Principal component analyses of the variance between microbiota compositions found placebo control and Av-CD291.2 posttreatment cohorts to overlap by both weighted (abundance) and unweighted (incidence) UniFrac dissimilarity score metrics with all pretreatment groups (Fig. 3A and B). For comparison, LD-fidaxomicin posttreatment samples did not overlap (90% inclusion zone) with placebo control or Av-CD291.2 posttreatment samples by incidence (Fig. 3B) and partially overlap (80% inclusion zone) by abundance (Fig. 3A). Comparison of abundance and incidence metrics obtained for posttreatment samples relative to pretreatment samples within each of the mice failed to detect a difference between placebo control and Av-CD291.2 cohorts (see Fig. S4B and C in the supplemental material); however, significant differences (*P* < 0.05) were observed between LD-fidaxomicin and the other cohorts, as expected (see Fig. S4B and C). Pairwise comparisons of whole microbiota dissimilarity by Adonis test failed to detect significant differences between Av-CD291.2 pre- and posttreatment samples or between Av-CD291.2 and placebo control posttreatment samples (see Table S1 in the supplemental material). Similar analyses did find LD-fidaxomicin posttreatment samples to differ (*P* < 0.05) by abundance and incidence metrics from LD-fidaxomicin pretreatment samples as well as Av-CD291.2 and placebo control posttreatment samples (see Table S1).

Analysis of the microbiome at various taxonomic levels indicated that few significant alterations to the composition of the gut microbiota occurred between pre- and posttreatment for the placebo control and Av-CD291.2 groups. At the family level, differences in *Rikenellaceae* (Fig. 3C; P < 0.01) and *Lactobacillaceae* (Fig. 3C and D; *P* < 0.05 and *P* < 0.01) were observed for Av-CD291.2 posttreatment samples relative to pretreatment samples; however, those differences were not specific to Av-CD291.2 treatment. *Rikenellaceae* in the Av-CD291.2 pretreatment samples were decreased relative to all other samples, while *Lactobacillaceae* were found to be reduced in all posttreatment cohorts, indicating that the duration of the sucrose and bicarbonate solution run-in (see Fig. S5B in the supplemental material) was not sufficient to allow the gut microbiota to fully adapt to the new carbohydrate-rich diet. For comparison, LD-fidaxomicin treatment resulted in significant differences by abundance and/or incidence UniFrac metrics (Fig. 3C and D) for the *Bacteriodales*: S24-7 (*Bacteroidetes*), *Ruminococcaceae* (*Firmicutes*), and *Lachnospiraceae* (*Firmicutes*). Those differences remained significant for the incidence metric when a false discovery rate correction was applied.

### Av-CD291.2 does not interfere with colonization resistance to C. difficile or vancomycin-resistant E. faecium (VREF).

**FIG 4:**
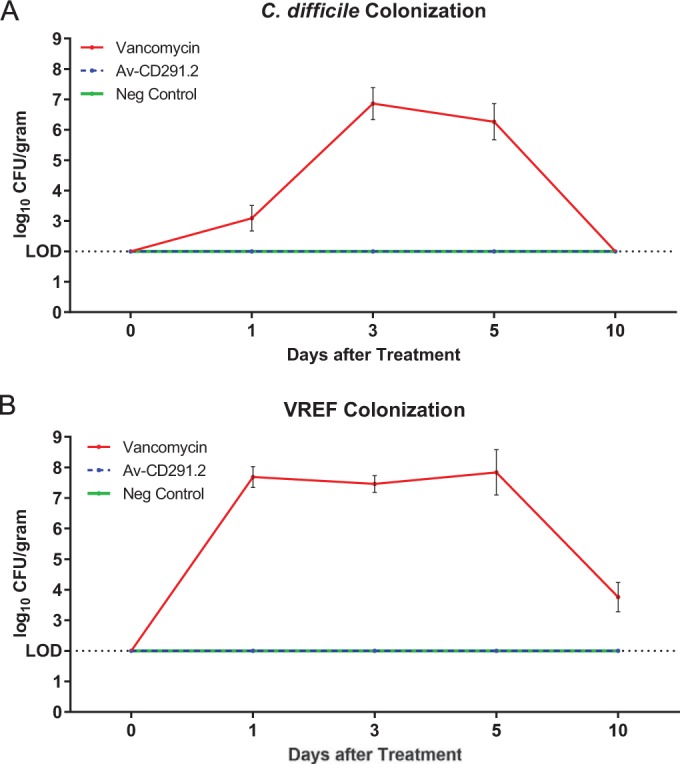
Oral administration of Av-CD291.2 in drinking water does not disturb colonization resistance to C. difficile (A) or vancomycin-resistant E. faecium (VREF) (B) in mice. Mice were administered Av-CD291.2 (2× the equivalent daily dose [mg/kg] in the microbiota study), vancomycin (37.5 mg/kg/day), or the placebo control for 4 days in the drinking water. Following a washout period, mice were inoculated with the BI/NAP1/027-type C. difficile strain or VREF C68 strain (10^4^ CFU) by oral gavage. Fecal samples were collected on days 1, 3, 5, and 10 postinoculation and assayed for C. difficile or VREF CFU. If pathogens were not detected in stool, the lower limit of detection (2 log_10_ CFU/g) was assigned. The standard errors of the means (SEM) for vancomycin-treated mice are shown.

The effective Av-CD291.2 dosing regimen from the prevention study was doubled and applied to normal healthy female mice (*n* = 10) to determine what impact Av-CD291.2 had on colonization resistance to C. difficile and the vancomycin-resistant Enterococcus faecium (VREF). Cohorts administered vancomycin (37.5 mg/kg per day) or the sucrose bicarbonate excipient solution (as in the microbiota study) were used as positive and negative controls, respectively. After 4 days of treatment and a washout period of 3 days when there was no detectable Av-CD291.2 in feces, mice were inoculated by oral gavage with 10^4^ CFU of C. difficile strain VA17 spores or 10^4^ CFU of VREF strain C68. VA17 is an epidemic North American pulsed-field gel electrophoresis type 1 (NAP1) C. difficile strain that has been used in previous mouse colonization studies and has MICs of 128 µg/ml and <1 µg/ml for clindamycin and vancomycin, respectively (30, 31). C68 is a clinical VanB VREF isolate that has also been used previously in mouse colonization studies (32). In the absence of antibiotic treatment, the resident microbiota of mice prevents establishment of colonization by VA17 and C68 (30–32). Mice with altered microbiota due to prior vancomycin administration became colonized with VA17 and VREF C68 as expected (33), with shedding levels reaching 10^8^ CFU/g of feces on day 5 (Fig. 4). No fecal shedding of VA17 or VREF C68 was detected (LOD = 100 CFU/g) for both the Av-CD291.2-treated mice and the excipient controls (Fig. 4). The results indicate that administration of Av-CD291.2 to mice with a healthy microbiota does not make them susceptible to C. difficile or VREF colonization once the treatment stops and suggest that administration of Av-CD291.2 to mice with a disrupted microbiota is unlikely to interfere with microbiota recovery and return of colonization resistance.

## DISCUSSION

The use of conventional antibiotics to treat bacterial diseases, such as CDI, comes with a paradox. Antibiotics are quite effective at eliminating a pathogen and reducing acute bacterial disease, but because of off-target effects, their very use can also make patients susceptible to reinfection or new infections by opportunistic pathogens that thrive in the absence of a diverse microbiota. New antibacterial agents that specifically target the pathogen with limited off-target effects are needed to escape this paradox. CDI is in desperate need of such agents. C. difficile is a leading cause of nosocomial infections worldwide (34, 35). As a nosocomial infection, CDI should be preventable, but incidence continues to rise (34, 36, 37). During CDI, the abundant shedding of spores impervious to most interventions makes it difficult to eradicate the pathogen from health care facilities and to limit person-to-person transmission (38, 39). Better hygienic practices and newer sporicides help to contain the spread of viable spores (7), but neither practice affects the primary source of spores—shedding from colonized patients. Treatment of CDI with antibiotics can cure the infection and reduce shedding, but the period between colonization and effective treatment provides time for the pathogen to shed spores, and relapses after “cure” are frequent. To effectively block transmission, one needs to prevent intestinal colonization or promptly decolonize the carrier. Filling that need requires a potent, highly specific prophylactic agent for C. difficile that eliminates the pathogen before it can proliferate and shed spores into the environment while preserving the healthy diversity of the gut microbiota and thus colonization resistance.

These data suggest that Avidocin-CDs may fulfill that function effectively. Av-CD291.2 was stable in drinking water and remained active during transit through the mouse GI tract. The robust oral efficacy of Av-CD291.2 in preventing BI-7 colonization and shedding during the spore transmission studies demonstrated the prophylactic potency of Avidocin-CD *in vivo* using an animal model reflective of human environmental exposure (24). The observation that BI-7 colonization and shedding occurred 3 days after cessation of Av-CD291.2 treatment was not unexpected since the gut microbiota had not had sufficient time to recover after being disrupted by clindamycin (26) and mice can harbor C. difficile spores in their fur for prolonged periods (40). The lack of detectable alterations to the diversity of gut microbiota and the maintenance of colonization resistance to C. difficile in naive mice attest to Avidocin-CD specificity and the absence of off-target effects among a wide array of bacteria. Based on these observations, it is likely that longer Avidocin-CD administration would have continued to protect from germinating spores without compromising or delaying the re-establishment of colonization resistance.

These findings provide a strong rationale for continuing to develop Avidocin-CDs and evaluate their clinical potential in managing human CDI. Our intent is to conduct proof-of-concept clinical studies with Av-CD291.2 as the product candidate. Because of the predominance of RT027 strains in many locations (~30% of CDI cases in the United States during 2011 through 2013) (2, 3) and the fact that this strain type is associated with increased disease severity and the highest rates of recurrence (2, 3, 6, 41), a stand-alone anti-RT027 agent, such as Av-CD291.2, will likely prove clinically important beyond proof-of-concept studies. A rapid and sensitive PCR-based diagnostic assay for ribotype 027 strains is readily available and makes it practical to rapidly identify asymptomatic patients colonized by or facilities burdened with Av-CD291.2-sensitive pathogens (42). For full coverage of C. difficile strain types, additional (or broader-spectrum) Avidocin-CDs will be needed. The successful fusion of the R-type bacteriocin scaffold to a newly identified C. difficile prophage RBP provides a template for their construction. The abundance of unique prophages in the C. difficile population and genome mining of intended target isolates have recently provided new Avidocin-CDs that kill other clinically relevant isolates. The resulting Avidocin-CDs could be given in a cocktail or deployed in conjunction with a point-of-intervention diagnostic that indicates the appropriate Avidocin-CD for the detected C. difficile strain or strains. The detection of a mixed infection (~10% of CDIs involve more than a single ribotype) would likely require deploying more than a single targeted Avidocin-CD (43, 44). More accurate diagnostic assays for Avidocin-CD deployment should be possible with the recent identification of the C. difficile surface target molecule for RBP binding (unpublished data).

The antibacterial properties observed for Av-CD291.2 suggest Avidocin-CDs could be effective therapeutic agents. However, we did not attempt to obtain supporting *in vivo* data for this application since several efficacious therapeutic options are readily available for treating acute CDI. Instead, we focused on CDI prophylaxis since it is a critical, unmet medical need, and the precisely targeted bactericidal properties of Avidocin-CD, as described herein, make these agents uniquely suited for prophylactic applications. Three prophylactic, clinical indications are apparent for such precision anti-C. difficile agents: (i) prevention of C. difficile colonization of high-risk individuals, (ii) prevention of acute, antibiotic-provoked CDI in asymptomatic C. difficile carriers, and (iii) prevention of recurrence in “cured” CDI patients. A prophylactic agent efficacious for any of these indications could reduce the morbidity, mortality, and health care costs associated with CDI. The preclinical data presented herein suggest Avidocin-CDs are a good candidate for preventing spore-mediated C. difficile colonization of individuals at high risk (i.e., option i). However, observations that approximately 30% of CDIs occur in antibiotic-treated asymptomatic carriers (45–47) make the second indication (i.e., option ii) a very feasible and potentially important option for Avidocin-based prophylaxis. The complete lack of BI-7 spore shedding during Av-CD291.2 administration in the spore transmission study reflects the *in vivo* potency of the Avidocin-CD and suggests that Avidocin-CDs could suppress the proliferation and spore shedding by vegetative C. difficile in asymptomatic carriers. Additional animal data will be needed to fully support Avidocin-CD use for this indication. Compared to preventing colonization of all individuals inside or entering a health care facility, the prevention of acute, antibiotic-provoked CDIs in asymptomatic C. difficile carriers would certainly require administration of Avidocin-CDs to fewer patients after screening with a point-of-intervention diagnostic to identify those carriers with Avidocin-CD291.2-sensitive C. difficle strains.

Two substantive considerations for human use of Avidocin-CDs are delivery mode and the emergence of resistance. In this study, sodium bicarbonate was used to buffer Av-CD291.2 from the acidity of the stomach and was necessary to deliver active Avidocin-CD to the lower GI tract in mice. The same need is expected for successful administration to humans and for which there are multiple commercially viable solutions. Other possible delivery methods include buffered solutions and enteric-coated capsules. The emergence of resistance is a reality for any antibacterial agent; Avidocin-CDs are no exception. We have observed the frequency of emerging resistance to Av-CD291.2 *in vitro* to be <10^−9^ (unpublished data). A common mechanism for the emergence of resistance to R-type bacteriocins is loss of the bacterial surface receptor (17, 19). However, because the receptor is unique to the target pathogen, Avidocin-CD agents will not promote the spread of drug resistance among the off-target, already insensitive organisms—an important, attractive property for a prophylactic agent.

With a better understanding of the human gut microbiota has come the knowledge that loss of microbial diversity results in vulnerability to many diseases (48–50). In spite of the extraordinary benefits of traditional antibiotics, the negative consequences of continuing to treat and mistreat bacterial diseases with antibiotics rife with off-target effects are beginning to look dire (9, 51). There is a need for “smart” antibacterial agents that can leverage rapid and accurate molecular diagnostic information at the point of intervention to guide precisely targeted protection from pathogens. With a low risk of spreading drug resistance to off-target organisms or disrupting protective microbiota, precision antibacterials can be deployed as safe prophylactics as well as therapeutics. Avidocin-CDs may serve as a prototype for this precision antibacterial platform.

## MATERIALS AND METHODS

### Experimental design.

The research objective was to determine whether an Avidocin-CD could prevent colonization by C. difficile in an established animal model and to measure the effect of the Avidocin-CD on the healthy gut microbiota and colonization resistance. The testing was done via controlled laboratory experiments using laboratory mice as research subjects. Animal care for *in vivo* studies was conducted under approved protocols in accordance with each institute’s guidelines (prevention study, Wellcome Trust Sanger Institute; microbiota study, ViviSource; colonization resistance study, Animal Care Committee of the Cleveland Veterans Affairs Medical Center). Mice were segregated according to gender (prevention study) and then randomly assigned to treatment groups. Investigators were not blinded to the animal treatment assignments. The treatment arms included Avidocin-CD for the prevention study, Avidocin-CD and a fidaxomicin control for the microbiota study, and Avidocin-CD and vancomycin in the colonization resistance study. All treatments were formulated in drinking water containing sodium bicarbonate and sucrose. Drinking water containing only sodium bicarbonate, and sucrose was used as a negative control for all three studies. For the prevention of colonization and colonization resistance studies, colonization was measured by the fecal presence or absence of the C. difficile strain used to contaminate the cages. The sample size for the prevention study (*n* = 20) was chosen by power analysis with the following parameters: α = 0.05, power = 80%, and df =1. For the microbiota studies, sequence analyses were performed on the DNA isolated from fecal samples before and after treatment. Based on the experience of the contracted vendor, the sample size for the microbiota study (*n* = 10) was chosen to minimize standard deviation values and increase the chances of detecting modest but significant alterations in the microbiota. Sample outliers were determined by comparison of pretreatment samples (*n* = 30) across multiple different parameters. No treatment samples were removed from the analyses. The sample size (*n* = 10) for the colonization resistance studies was based on the effect size from previous published research (52).

### Bacterial strains, plasmids, and diffocin constructs. (i) C. difficile and E. faecium (VREF) strains.

The C. difficile strains described in this article are human isolates. Each isolate is listed with source and related information in Table S2 in the supplemental material. Clinical isolates purchased from the R. M. Alden Research Lab, Culver City, CA, were obtained from a large North American clinical study during 2006 to 2008 (53). Genomic DNA from C. difficile isolates ATCC 43593 and R20291 (GenBank accession no. FN545816.1) was extracted and purified with MasterPure Gram-positive DNA purification kits (Illumina, San Diego, CA) and used to clone diffocin gene clusters and prophage RBP genes. VREF strain C68 is a human VanB-type isolate of E. faecium that has been used in previous mouse model studies (52).

### (ii) Diffocin/Avidocin-CD production strains and plasmids.

Diffocin gene clusters were cloned and expressed in B. subtilis. All B. subtilis production strains used in this study are listed in Table S2 in the supplemental material and derive from strain BDR11 (provided D. Rudner, Harvard University), which in turn is a derivative of strain PY79 (GenBank accession no. NC_022898.1) (54, 55). All oligonucleotides and plasmids used to construct B. subtilis production strains are listed in Table S3 in the supplemental material. To improve diffocin yield, we removed several genes from BDR11 via a marker-free deletion method published by Liu et al. (56). First, the entire PBSX prophage was eliminated (ΔPBSX) to create strain BDG9, as previously described (14). Next, we deleted the *spoIIGA* gene from BDG9 to create a nonsporulating strain, BDG77 (ΔPBSX; Δ*spoIIGA*). To produce diffocins in these strains, diffocin gene clusters were introduced into the chromosome at the *amyE* locus by double homologous recombination using integration vectors derived from the pDR111 plasmid (provided by D. Rudner, Harvard University). Each integration vector contained short *amyE* sequences flanking a diffocin gene cluster adjacent to an antibiotic resistance marker (*cat* or *spec*). Due to the ~20-kb size, the diffocin gene cluster was cloned into the integration vector using multiple DNA fragments via the Gibson assembly method (57). For diffocin-43593, DNA fragments spanning *orf1359* to *rtbG*, amplified by PCR with primers AV1289 and oDG376, *rtbH* to the proximal portion of *rtbM*, amplified by PCR with primers oDG393 and oDG15, and the distal portion of *rtbM* to *rtbO*, amplified by PCR with primers oDG392 and AV1289, were used to create pDG636. For modified diffocins containing the phi027 prophage PtsM protein, DNA fragments containing the diffocin *rtbM* gene in plasmid pDG579 were replaced with DNA fragments spanning *rtbH* to the proximal portion of the diffocin *bpar* gene (amplified with primers oDG591 and oDG602) and either two DNA fragments (distal portion of the phi027 bpar gene, *ptsL*, to the distal end of the *ptsM* gene, amplified with primers oDG603 and oDG604, and diffocin *rtbN* to *rtbO*, amplified with primers oDG605 and oDG590) to create pDG721 encoding Av-CD291.1, or one DNA fragment (distal portion of the phi027 *ptsN* gene to the phi027 *ptsO* gene, amplified with primers oDG603 and oDG785) to create pDG779 encoding Av-CD291.2. Transformation of pDG636 into BDG9 created strain BDG59 (diffocin-43593). Transformation of pDG721 into BDG77 created strain BDG127 (Av-CD291.1). Transformation of pDG779 into BDG9 created strain BDG189 (Av-CD291.2). Creation of BDG45, which produces diffocin-4, was previously described (14).

### Diffocin/Avidocin-CD expression and *in vitro* testing. (i) Diffocin/Avidocin-CD expression.

Seed cultures (tryptic soy broth [BD-Difco]) for production strains were back-diluted 1:50 in 2× Terrific Broth (BD-Difco) and incubated at 37°C with shaking (225 rpm). At an optical density at 600 nm (OD_600_) of ~0.3/cm, cultures were shifted to 28°C. At an OD_600_ of ~1.0/cm, hydrogen peroxide (5 mM final concentration) was added to induce a SOS response and diffocin expression. Hydrogen peroxide-treated cultures were maintained at 28°C overnight with shaking (225 rpm), after which cells were harvested at 7,000 × *g* for 30 min.

### (ii) Diffocin/Avidocin-CD preparations.

The method used was a modification of large-scale phage purification protocol that relies on polyethylene glycol (PEG) to concentrate and purify large, asymmetrical particles from bacterial cell lysates (58). First, cell pellets were resuspended in 20 mM HEPES (1:6 cell mass-to-buffer volume) containing 1 mg/ml egg white lysozyme and 12 U of Benzonase/g of cell mass, and incubated with mild agitation at room temperature for 60 min to lyse intact cells and digest DNA. Cell lysates were then centrifuged for 60 min at 30,000 × *g* to remove cellular debris. The resulting clarified lysates were then combined with culture supernatants collected earlier. PEG-8000 (10% [wt/wt] final concentration) and NaCl (0.5 M final concentration) were added to the combined solution, and the mixture was incubated overnight at 4°C. Precipitates containing diffocin were pelleted by centrifugation at 16,000 × *g* for 45 min. Pellets were resuspended in HN50C (20 mM HEPES [pH 7.4], 50 mM NaCl, 10 mM CaCl_2_), and insoluble debris was removed by centrifugation at 30,000 relative centrifugal force (RCF) for 30 min. Clarified resuspensions were then ultracentrifuged at 90,000 RCF for 3 h to further purify the diffocins. The resulting diffocin-containing pellets were resuspended by careful repeated pipetting in HN50C at 0.5% of the original culture volume. The final diffocin solution was clarified by centrifugation at 16,000 RCF for 30 min and passed through a 0.45-µm-pore polyethersulfone (PES) filter (VWR) and stored at 4°C or −80°C.

### (iii) *In vitro* spot bioassays for R-type bacteriocin killing activity.

Bactericidal activity was assayed by the semi-quantitative spot method or the titration method as described by Gebhart et al. (14).

### Prevention study in mouse model of C. difficile spore transmission. (i) Fecal sample processing.

Fresh fecal pellets were collected and weighed. HN50C containing Complete protease inhibitor cocktail (Roche) was added to a final concentration of 0.1 mg/ml. A pellet pestle (Kontes) was used to disrupt the pellets in solution and create a homogenate. The resulting homogenates were then centrifuged in a microcentrifuge at 14,000 RCF for 1 min to pellet debris. Clarified homogenates were filtered through a 0.45-µm-pore PES filter (VWR) and stored at 4°C before assaying for diffocin/Avidocin-CD killing activity.

### (ii) Enteric pharmacokinetic studies.

For oral gavage studies, 6-week-old C57BL/6 female mice were fasted for 2 h and then given an oral gavage containing a cocktail containing either diffocin-4 or Av-CD291.2 (~100 µg or 5 × 10^11^ killing units [KU]) formulated in 1% NaHCO_3_. Thirty minutes after gavage, food was returned to the mice. Fresh fecal pellets were collected at several time points after the gavage, processed, and assayed for bactericidal activity as described above. For administration in drinking water, Av-CD291.2 was formulated in an excipient solution containing 4% sucrose and 1% NaHCO_3_ at a concentration of 60 µg/ml (3 × 10^11^ KU/ml). Addition of sucrose (4%) to the drinking water increased water consumption and reduced the diurnal variation observed with unsweetened water (23). Graduated drinking tubes (22, 59) were used to deliver the drinking solution and measure daily consumption by each animal. Fecal pellets were collected at 4, 8, 12, 24, and 28 h after the start of Av-CD291.2 administration, processed, and assayed for bactericidal activity as described above.

### (iii) Prevention of colonization in mouse model of spore transmission.

The prevention-of-colonization experiments were an adaptation of a mouse model for environmental spore transmission of C. difficile (24). A schematic of the experimental timeline is shown in Fig. S5A in the supplemental material. In the study, C. difficile spores purified from BI-7 (RT027) cultures were resuspended in a 70% ethanol solution (10 ml) and spread across the floors of sterile mouse cages (no bedding) at a final spore density of 13 CFU/cm^2^. The cages were allowed to dry in laminar flow biosafety cabinets for 4 h after spore spreading. Four- to 5-week-old male (*n* = 20) and female (*n* = 20) C57BL/6 mice were placed in the contaminated cages (10 per cage, segregated by gender) for 1.5 h and then transferred to individual sterile isolator cages via a laminar flow biosafety cabinet. For treatment, placebo (excipient solution) and Av-CD291.2 at 25 µg/ml (1.1 × 10^11^ KU/ml) of excipient solution were administered via graduated drinking tube to identical cohorts (10 male and 10 female) beginning 4 h prior to spore exposure and continuing through day 4 of the study. Diffocin and placebo drinking solutions were replaced with fresh solutions daily. The amount of drinking solution consumed daily by each mouse was recorded and used to calculate dosage. On day 2 after spore exposure, clindamycin (200 mg/ml) was added to the drinking solution for all mice to promote C. difficile colonization and super shedding. On day 4 after spore exposure, administration of Av-CD291.2 and placebo ceased, and fresh fecal pellets were collected and homogenized in phosphate-buffered saline (PBS). Plating of the homogenates on multiple selective Brazier’s agar plates containing 0.5% taurocholate was performed within 60 min of isolation.

To differentiate BI-7 (clindamycin-resistant) from potential C. difficile contaminants, 20 mg/ml clindamycin was added to one set of plates. Reconstruction studies with fecal homogenates spiked with fresh Av-CD291.2 and C. difficile spores confirmed that the Avidocin-CD levels found in feces after administration in drinking water were insufficient to inhibit culturing of germinating spores on C. difficile selective agar plates containing taurocholate (data not shown). On day 7 after spore exposure, fresh fecal pellets were collected again and cultured for C. difficile as described above. Statistical analyses of the colonization rate were performed with Fisher’s exact test (GraphPad), and *P* values of <0.05 are reported.

### Microbiota study in naive mice. (i) Animal procedures.

A schematic of the experimental timeline is shown in Fig. S5B in the supplemental material. After a 4-day acclimation, 6-week-old female C57BL/6 mice (*n* = 30) were individually caged and administered drinking water containing excipients (placebo: 4% sucrose and 1% NaHCO_3_). After 3 days, fresh fecal samples from each animal were collected via a clean catch method (in which pellets are expressed directly into a sterile vessel) and stored at −80°C. This time point is referred to as day −1 or “pretreatment.” On the next day (day 0), the mice were divided into 3 groups of 10 mice each, caged individually, and began receiving by graduated drinking tube either placebo (excipient solution; *n* = 10), Av-CD291.2 in excipient solution, or low-dose fidaxomicin (LD-fidaxomicin; 2-µg/ml final concentration) in excipient solution. The dosage and duration of Av-CD291.2 and placebo treatments were identical to those used in the prevention-of-colonization study. The daily consumption of drinking solution was recorded for each mouse. The average daily dose of fidaxomicin was 0.8 mg/kg body weight, which is on the lower end of the effective dose range found for hamsters (0.2 mg/kg to 5 mg/kg) (29) and far below the recommended 5-mg/kg human daily dose (28). On day +2, fecal samples from Av-CD291.2-treated mice were collected, processed, and assayed for Av-CD291.2 activity via spot bioassays for bactericidal activity (as described above) to ensure that Av-CD291.2-treated mice were receiving active diffocin during the study. On day +4, fecal samples designated “posttreatment” were again collected via a clean catch method and stored at −80°C. Fecal samples were shipped to Second Genome, Inc. (South San Francisco, CA), for further processing. Total genomic DNA (gDNA) was extracted from samples using Powermag DNA isolation kit (Mobio) as per the manufacturer’s instructions. All extractions were performed in a pre-PCR clean room.

### (ii) 16S ribosomal RNA gene sequencing.

Purified gDNA from pre- and posttreatment samples was then prepared by Second Genome, Inc., for sequencing and analysis. Fusion primers containing indexing bar codes specific to each sample were used to amplify bacterial 16S V4 rRNA gene regions. The barcoded PCR products from each sample were pooled for sequencing on the Illumina Miseq with 250-bp paired-end reads. A total of 120,136 to 348,656 reads per sample were obtained. QIIME and custom scripts were used to quality filter and demultiplex the sequencing reads (60). Assignment of taxonomic classification for each resulting sequence was performed as described by Xuan et al. (49).

### (iii) Data analyses.

All profiles were intercompared in a pairwise fashion to determine a dissimilarity score across the entire community for all samples. The weighted UniFrac dissimilarity score utilizes the taxon abundance differences across samples but employs a pairwise normalization by dividing the sum of differences by the sum of all abundances (61). The unweighted UniFrac score considers only the presence or absence (incidence) of taxa (62, 63). Calculation of community-wide dissimilarity measures was based on 120,136 selected sequences. For OTU abundance, samples were normalized to 1 million counts. Principal component analyses and Adonis tests were used to identify significant overall difference in microbiota community structure. Pretreatment sample outliers (1 per cohort; circled in Fig. 3A and B) identified through multiple comparisons (weighted UniFrac dissimilarity, UniFrac measure, total OTU counts, and principal component analyses) were removed from further analyses. Student’s *t* test and false discovery rate correction were used to identify family taxa that differed between cohorts.

### (iv) Statistical analysis.

Statistical analyses were performed as indicated in each method section or figure legend. *P* values of <0.05 were considered significant.

### Mouse model of colonization resistance.

Sixty female CF-1 mice weighing 25 to 30 g (Harlan Sprague-Dawley, Indianapolis, IN) were housed in individual micro-isolator cages. Mice (10 per group) received Av-CD291.2, vancomycin (37.5 mg/kg per day), or placebo in water containing sucrose (4%) and sodium bicarbonate (1%) for 4 days. The drinking water was administered using a calibrated fluid administration device that replaced the usual water bottle in the cage (22). The graduated devices were refilled daily. *In vitro* spot bioassays for bactericidal activity were used to confirm that Av-CD291.2-treated mice shed active Avidocin-CD in feces during treatment. Three days after discontinuation of treatment, mice were challenged by oral gavage with 10^4^ CFU of C. difficile VA17 spores or 10^4^ CFU of E. faecium C68 (VREF). *In vitro* spot bioassays for bactericidal activity were used to confirm that Av-CD291.2-treated mice were no longer shedding active bacteriocin prior to challenge. Fresh stool specimens were collected 1, 3, 5, and 10 days after gavage and used to measure the concentration of pathogens by plating serially diluted samples on selective agars. Prereduced cycloserine-cefoxitin-brucella agar containing 0.1% taurocholic acid and 5 mg/ml lysozyme (CDBA) and Enterococcosel agar (Becton, Dickinson, Sparks, MD) containing 20 µg/ml of vancomycin were used for C. difficile and VREF, respectively (30, 50). Colonization resistance was deemed intact at a given time point if there was no significant increase in concentration of the pathogens in the stool of Av-CD291-treated mice in comparison to that from the placebo control mice.

## SUPPLEMENTAL MATERIAL

Dataset S1 Total *C. difficile* shedding (CFU/g feces) during the prevention-of-colonization study. Download Dataset S1, XLSX file, 0.01 MB

Dataset S2 Presence and absence of detected OTU in the fecal microbiota study. Download Dataset S2, XLSX file, 0.9 MB

Figure S1  Schematic of a diffocin and its mechanism of action. Diffocin is shown in the unbound and bound states on the C. difficile cell surface. RBP, receptor binding protein; BPAR, baseplate attachment region. Download Figure S1, PDF file, 0.2 MB

Figure S2  Stability of R-type bacteriocins targeting RT027 strains. *In vitro* spot killing bioassays on strain R20291 (RT027) are shown. Preparations of diffocin-43593, Av-CD291.1, and Av-CD291.2 were isolated from B. subtilis production strains and then immediately spotted (day 0) or stored for 3 days before spotting on a soft agar lawn containing the target strain. Dark zones of clearance indicate killing. Download Figure S2, PDF file, 0.1 MB

Figure S3  Spores are not killed by Avidocin-CDs *in vitro*. Approximately 50,000 CFU of R20291 spores were incubated with a 100-fold excess of Av-CD291.2 (5 × 10^6^ KU) to CFU for 60 min in triplicate. After incubation, the samples were serially diluted and plated on brucella agar plates containing 0.1% taurocholate. A mock incubation with PBS for 60 min (and then heat treatment at 65°C to kill nonspores) was used as a control. No difference in viable CFU counts was observed. Similar experiments with R20291 vegetative cells (using up to 10^9^ cells) and a 100-fold of excess Av-CD291.2 (up to 10^11^ KU) resulted in no CFU being recovered. Download Figure S3, PDF file, 0.001 MB

Figure S4  Additional microbiota analyses. (A) Alpha diversity pre- and posttreatment. The mean numbers of OTU observed for each cohort pre- and posttreatment were calculated. No significant differences were observed pre- and posttreatment for any cohort. Error bars indicate standard deviation for each condition (B and C). Comparison of microbiota change within each mouse after treatment shows no significant difference between treatment with Av-CD291.2 and treatment with the placebo control. (B) The variance between microbial communities posttreatment relative to pretreatment within each mouse was assessed by averaging the weighted UniFrac distances (relative change in OTU abundances) for each cohort. The mean for each treatment is indicated by a red bar and labeled. Error bars indicate standard deviations. Student’s *t* tests were used to compare results. *P* values of <0.05 are indicated. Abundance variation after LD-fidaxomicin treatment was significantly different from Av-CD291.2 or placebo control treatments. (C) The plot is the same as in panel B, except the average unweighted UniFrac distance for each cohort was assessed. Incidence variation after LD-fidaxomicin treatment was significantly different from that of Av-CD291.2 or placebo control treatments. Download Figure S4, PDF file, 0.1 MB

Figure S5  Timeline schematics for prevention of colonization and microbiota studies in mice. (A) Av-CD291.2 efficacy study in mice exposed to spores from the BI-7 strain. Spore exposure and fecal sampling time points are depicted by arrows. The durations of the Av-CD291.2, placebo, and clindamycin treatments are indicated by parentheses. (B) Microbiota study in naive mice. Download Figure S5, PDF file, 0.1 MB

Table S1  Summary of *P* value results for Adonis tests. Table S1, PDF file, 0.2 MB

Table S2  Strain list. Table S2, PDF file, 0.2 MB

Table S3  Oligonucleotide and plasmid lists. Table S3, PDF file, 0.2 MB
