# Supplementary figures and images for: A Modified R-Type Bacteriocin Specifically Targeting Clostridium difficile Prevents Colonization of Mice without Affecting Gut Microbiota Diversity
Source: mBio. 2015 Mar 24;6(2):e02368-14. doi: 10.1128/mBio.02368-14 (PMC4453579; doi:10.1128/mBio.02368-14)

Fig. S1

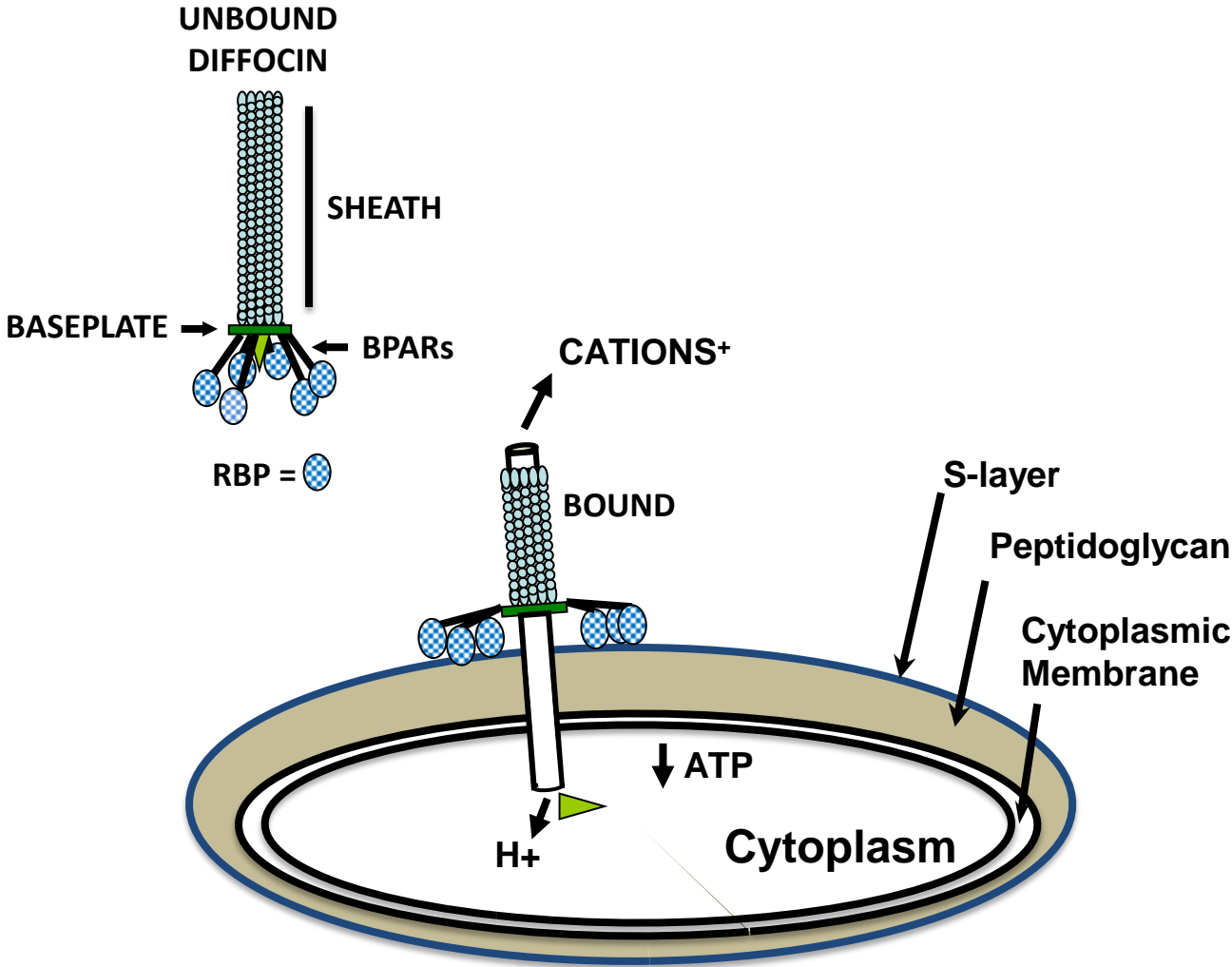

Supplement: Figure S1 — Schematic of a diffocin and its mechanism of action. Diffocin is shown in the unbound and bound states on the C. difficile cell surface. RBP, receptor binding protein; BPAR, baseplate attachment region. Download [file mbo002152236sf1.pdf]

Fig. S2

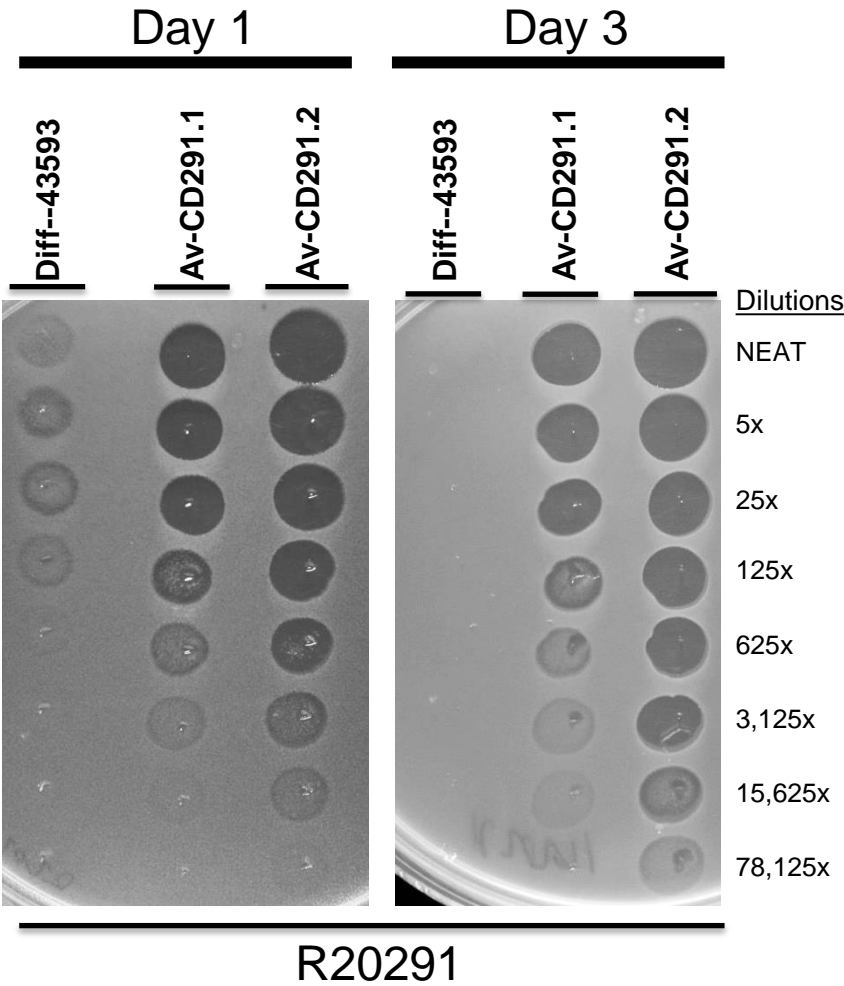

Supplement: Figure S2 — Stability of R-type bacteriocins targeting RT027 strains. In vitro spot killing bioassays on strain R20291 (RT027) are shown. Preparations of diffocin-43593, Av-CD291.1, and Av-CD291.2 were isolated from B. subtilis production strains and then immediately spotted (day 0) or stored for 3 days before spotting on a soft agar lawn containing the target strain. Dark zones of clearance indicate killing. Download [file mbo002152236sf2.pdf]

Fig. S3

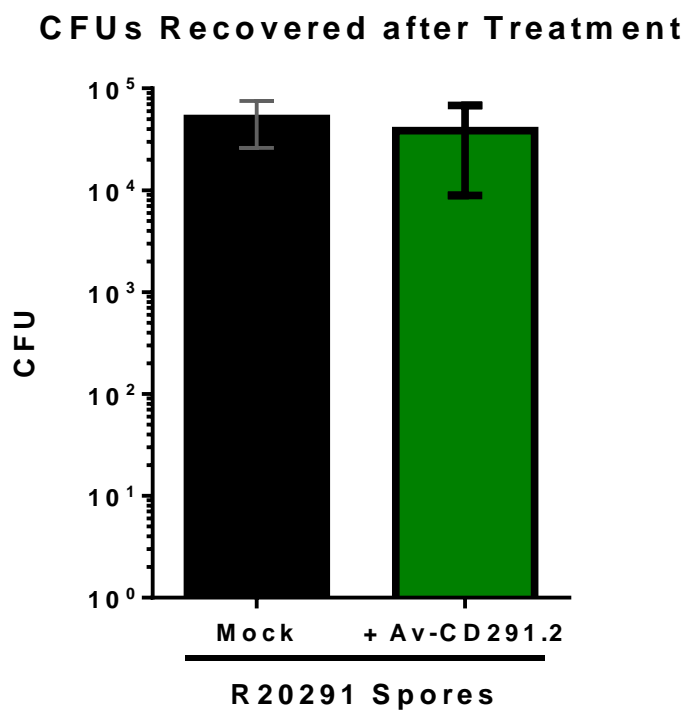

Supplement: Figure S3 — Spores are not killed by Avidocin-CDs in vitro. Approximately 50,000 CFU of R20291 spores were incubated with a 100-fold excess of Av-CD291.2 (5 × 10^6^ KU) to CFU for 60 min in triplicate. After incubation, the samples were serially diluted and plated on brucella agar plates containing 0.1% taurocholate. A mock incubation with PBS for 60 min (and then heat treatment at 65°C to kill nonspores) was used as a control. No difference in viable CFU counts was observed. Similar experiments with R20291 vegetative cells (using up to 109 cells) and a 100-fold of excess Av-CD291.2 (up to 1011 KU) resulted in no CFU being recovered. Download [file mbo002152236sf3.pdf]

Fig. S4

A

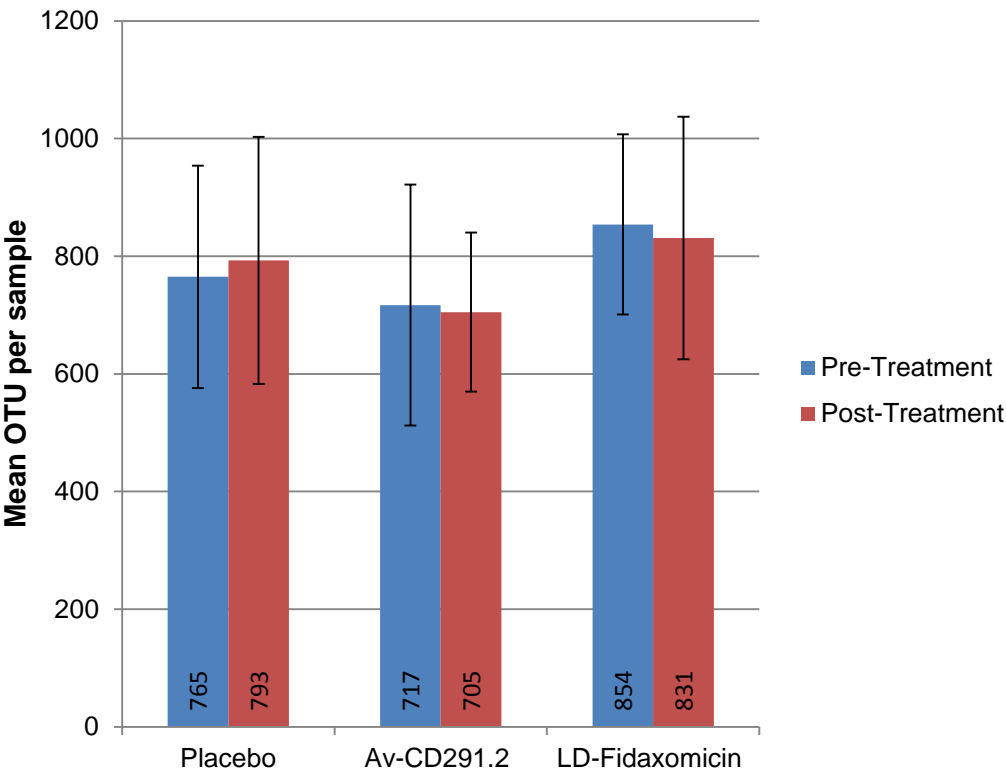

B

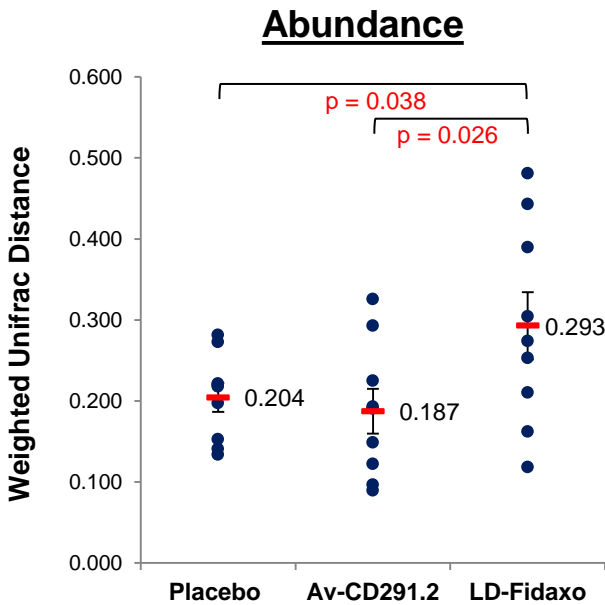

C

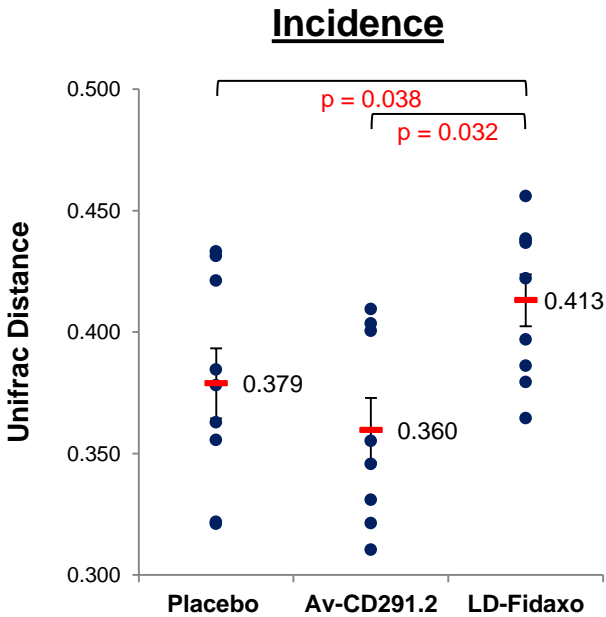

Supplement: Figure S4 — Additional microbiota analyses. (A) Alpha diversity pre- and posttreatment. The mean numbers of OTU observed for each cohort pre- and posttreatment were calculated. No significant differences were observed pre- and posttreatment for any cohort. Error bars indicate standard deviation for each condition (B and C). Comparison of microbiota change within each mouse after treatment shows no significant difference between treatment with Av-CD291.2 and treatment with the placebo control. (B) The variance between microbial communities posttreatment relative to pretreatment within each mouse was assessed by averaging the weighted UniFrac distances (relative change in OTU abundances) for each cohort. The mean for each treatment is indicated by a red bar and labeled. Error bars indicate standard deviations. Student’s t tests were used to compare results. P values of <0.05 are indicated. Abundance variation after LD-fidaxomicin treatment was significantly different from Av-CD291.2 or placebo control treatments. (C) The plot is the same as in panel B, except the average unweighted UniFrac distance for each cohort was assessed. Incidence variation after LD-fidaxomicin treatment was significantly different from that of Av-CD291.2 or placebo control treatments. Download [file mbo002152236sf4.pdf]

Fig. S5

A.

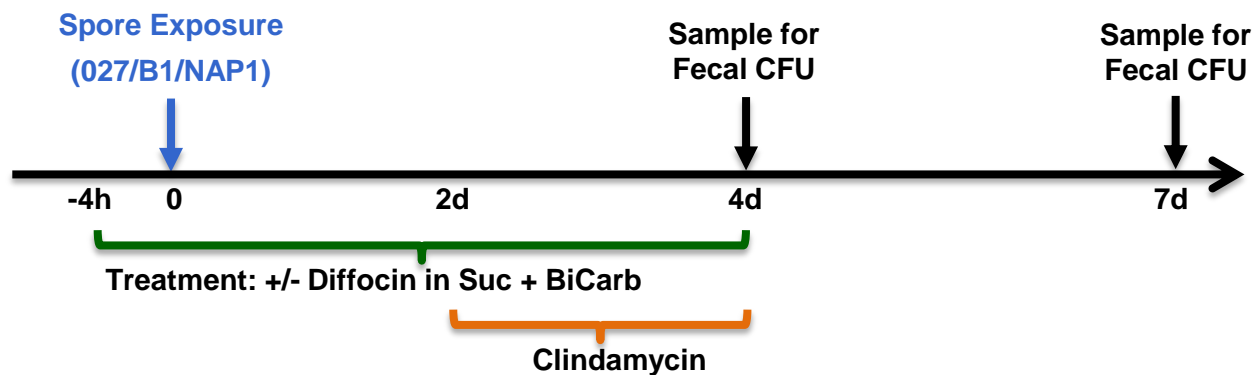

B.

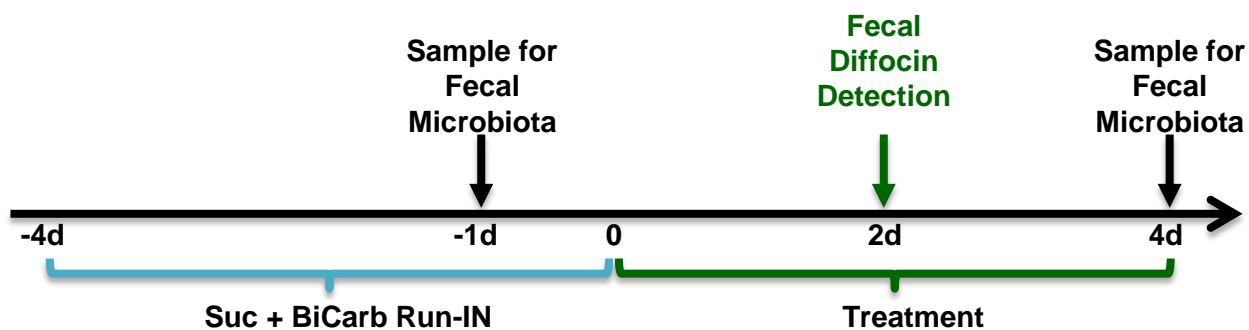

Supplement: Figure S5 — Timeline schematics for prevention of colonization and microbiota studies in mice. (A) Av-CD291.2 efficacy study in mice exposed to spores from the BI-7 strain. Spore exposure and fecal sampling time points are depicted by arrows. The durations of the Av-CD291.2, placebo, and clindamycin treatments are indicated by parentheses. (B) Microbiota study in naive mice. Download [file mbo002152236sf5.pdf]
