# Supplementary material for: A Modified R-Type Bacteriocin Specifically Targeting Clostridium difficile Prevents Colonization of Mice without Affecting Gut Microbiota Diversity
Source: mBio. 2015 Mar 24;6(2):e02368-14. doi: 10.1128/mBio.02368-14 (PMC4453579; doi:10.1128/mBio.02368-14)
Supplement: Table S1 — Summary of P value results for Adonis tests. [file mbo002152236st1.pdf]

Table S1. Summary of p-value results for Adonis tests. Adonis tests were performed on pair-wise comparisons between treatment cohorts.

Pair-Wise Comparisons			Adonis Test (p-value)	
			**Abundance**	**Incidence**
	Av-CD291.2 Pre-Treatment	Av-CD291.2 Post-Treatment	0.136	0.48
	Placebo Pre-Treatment	Placebo Post-Treatment	0.303	0.097
	LD-Fidaxomicin Pre-Treatment	LD-Fidaxomicin Post-Treatment	**0.02**	**0.001**
	Placebo Post-Treatment	Av-CD291.2 Post-Treatment	0.229	0.107
	Av-CD291.2 Post-Treatment	LD-Fidaxomicin Post-Treatment	**0.005**	**0.001**
	LD-Fidaxomicin Post-Treatment	Placebo Post-Treatment	**0.042**	**0.003**

The p-values < 0.05 are highlighted in **red**.
