# Supplementary material for: A Modified R-Type Bacteriocin Specifically Targeting Clostridium difficile Prevents Colonization of Mice without Affecting Gut Microbiota Diversity
Source: mBio. 2015 Mar 24;6(2):e02368-14. doi: 10.1128/mBio.02368-14 (PMC4453579; doi:10.1128/mBio.02368-14)
Supplement: Table S2 — Strain list. [file mbo002152236st2.pdf]

Table S2. Strain List.

Species	Strain Designation	Ribo-type	Source	Comment – Isolation - Reference
Bacillus subtilis	BDR11		David Rudner	Parent of *B. subtilis* strains
Bacillus subtilis	BDG9		Gebhart, *et al*. 2012	ΔPBSX
Bacillus subtilis	BDG45		Gebhart, *et al*. 2012	Diffocin-4 producer
Bacillus subtilis	BDG59		this paper	Diffocin-43593 producer
Bacillus subtilis	BDG77		this paper	ΔPBSX, ΔSpoIIGA
Bacillus subtilis	BDG127		this paper	Av-CD291.1 producer
Bacillus subtilis	BDG189		this paper	Av-CD291.2 producer
Clostridium difficile	19103	001	R.M. Alden Research	N. America/2006-8 / Louie *et al*., 2011
Clostridium difficile	19135	001	R.M. Alden Research	N. America / 2006-8 / Louie *et al*., 2011
Clostridium difficile	BI-9	001	Trevor Lawley	Gerding Collection / He *et al*., 2010
Clostridium difficile	Liv24	001	Trevor Lawley	Liverpool UK / 2009
Clostridium difficile	19137	015	R.M. Alden Research	N. America / 2006-8 / Louie *et al*., 2011
Clostridium difficile	TL174	015	Trevor Lawley	Cambridge UK / 2004
Clostridium difficile	CF5	017	Trevor Lawley	Belgium /1995 / He *et al*., 2010
Clostridium difficile	M68	017	Trevor Lawley	Ireland / 2006 / He *et al*., 2010
Clostridium difficile	19104	027	R.M. Alden Research	N. America / 2006-8 / Louie *et al*., 2011
Clostridium difficile	19113	027	R.M. Alden Research	N. America / 2006-8 / Louie *et al*., 2011
Clostridium difficile	19124	027	R.M. Alden Research	N. America / 2006-8 / Louie *et al*., 2011
Clostridium difficile	19126	027	R.M. Alden Research	N. America / 2006-8 / Louie *et al*., 2011
Clostridium difficile	19129	027	R.M. Alden Research	N. America / 2006-8 / Louie *et al*., 2011
Clostridium difficile	19136	027	R.M. Alden Research	N. America / 2006-8 / Louie *et al*., 2011
Clostridium difficile	19138	027	R.M. Alden Research	N. America / 2006-8 / Louie *et al*., 2011
Clostridium difficile	19139	027	R.M. Alden Research	N. America / 2006-8 / Louie *et al*., 2011
Clostridium difficile	19143	027	R.M. Alden Research	N. America / 2006-8 / Louie *et al*., 2011
Clostridium difficile	19153	027	R.M. Alden Research	N. America / 2006-8 / Louie *et al*., 2011
Clostridium difficile	19684	027	R.M. Alden Research	N. America / 2006-8 / Louie *et al*., 2011
Clostridium difficile	20068	027	R.M. Alden Research	N. America / 2006-8 / Louie *et al*., 2011
Clostridium difficile	BI-7	027	Trevor Lawley	Portland, OR / 2003 / He *et* al., 2010
Clostridium difficile	CD196	027	Trevor Lawley	Paris, Fr /1985 / He *et* al., 2010
Clostridium difficile	R20291	027	Trevor Lawley	Aylesbury, UK / 2006 / He *et* al., 2010
Clostridium difficile	VA17	027	Curtis Donskey	Cleveland, OH/ 2005 / Pultz *et* al., 2005
Clostridium difficile	19108	053	R.M. Alden Research	N. America / 2006-8 / Louie *et al*., 2011
Clostridium difficile	19110	053	R.M. Alden Research	N. America / 2006-8 / Louie *et al*., 2011
Clostridium difficile	19147	053	R.M. Alden Research	N. America / 2006-8 / Louie *et al*., 2011
Clostridium difficile	ATCC 43255	087	ATCC	Toxinotype 0 ref strain / Hammond *et al*., 1995
Clostridium difficile	ATCC 43593	060	ATCC	Belgium / 1984 / Delmee *et al*., 1985
Enterococcus faecium	(VRE) C68		Curtis Donskey	Cleveland / 1996 / Donskey *et al*., 1999
