# Supplementary material for: A Modified R-Type Bacteriocin Specifically Targeting Clostridium difficile Prevents Colonization of Mice without Affecting Gut Microbiota Diversity
Source: mBio. 2015 Mar 24;6(2):e02368-14. doi: 10.1128/mBio.02368-14 (PMC4453579; doi:10.1128/mBio.02368-14)
Supplement: Table S3 — Oligonucleotide and plasmid lists. [file mbo002152236st3.pdf]

Table S3.

A. Oligonucleotide List

Oligonucleotide Name	Sequence
AV1288	Gaagaaagagatgggactcgagatg
AV1289	tatacatctcgagtcccatctctttc
oDG15	tccttcggcgcgcctcaaatttaagcttaactcc
oDG376	ttcctcgcggccgcctgttaagccagttaaaattactac
oDG392	gattgcatgcgagtgagtag
oDG393	ctactcactcgcatgcaatc
oDG590	ggcgctcaggatccggcgcgcctcaaatttaagctt
oDG591	tctggaaataatctagaactttcgaagatatatctagaactttcaaagatg
oDG602	gcttattttagcatctaaatcttgaatatccttttgagttaca
oDG603	taactcaaaaggatattcaagatttagatgctaaaataagca
oDG604	aattttcacacctccattttaaataaatttaatagcataccatt
oDG605	ggtatgctattaaatttatttaaaatggaggtgtgaaaattgt
oDG785	ggcgctcaggatccggcgcgccctatatagttggttctgctcc

B. Plasmid List

Plasmid Name	Source	Description
pDG636	Gebhart, *et al*. 2012	Construct for Diffocin -4 production
pDG636	this paper	Construct for Diffocin-43593 production
pDG721	this paper	Construct for Av-CD291.1 production
pDG779	this paper	Construct for Av-CD291.2 production
